# Supplementary material for: Development of Single-Molecule Electrical Identification Method for Cyclic Adenosine Monophosphate Signaling Pathway
Source: Nanomaterials (Basel). 2021 Mar 19;11(3):784. doi: 10.3390/nano11030784 (PMC8003578; doi:10.3390/nano11030784)
Supplement: Supplementary file 1 [file nanomaterials-11-00784-s001.pdf]

nanomaterials

MDPI

Supplementary Materials

Development of Single-Molecule Electrical Identification Method for Cyclic Adenosine Monophosphate Signaling Pathway

Yuki Komoto [1,2], Takahito Ohshiro [1] and Masateru Taniguchi [1,*]

[1] Institute of Science and Industrial Research, Osaka University, 8-1 Mihogaoka, Ibaraki, Osaka 567-0047, Japan; komoto@sanken.osaka-u.ac.jp (Y.K.); toshiro@sanken.osaka-u.ac.jp (T.O.)
[2] Artificial Intelligence Research Center, The Institute of Scientific and Industrial Research, Osaka University, 8-1 Mihogaoka, Ibaraki, Osaka 567-0047, Japan
* Correspondence: taniguti@sanken.osaka-u.ac.jp; Tel.: +81-6-6879-8447

S1. Current-Time Profiles for Cyclic AMP

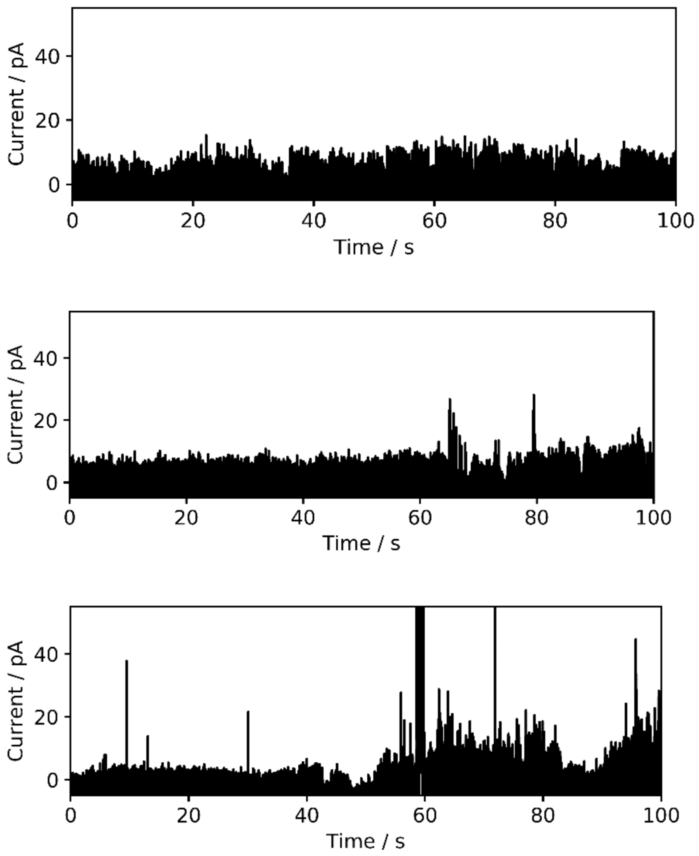

Figure S1. Current–time profiles for signals obtained cAMP aqueous solution.

S2. Distribution of the first component for AMP and cAMP

It is essential to use multidimensional data as in our method. In fact, the 13-dimensional feature vector we used shows differences. The features we used were standardized so that the mean is 0 and the standard deviation is 1. Then the first principal component (PC1) of the result of principal component analysis (PCA) is shown in the figure. In the figure, we can clearly see the difference. The first principal component in this case is the vector represented by 0.29I_p + 0.05I_d + 0.09I_{ave} + 0.23S_1 + 0.30S_2 + 0.31S_3 + 0.31S_4 + 0.30S_5 +

0.30S_6 + 0.31S_7 + 0.30S_8 + 0.29S_9 + 0.33S_{10}. The large overlap arises because the 13-dimensional feature space was reduced to one dimension for the sake of illustration. 13-dimensional space cannot be illustrated, but the overlap is smaller in 13-dimensional space. Although the results of the principal component analysis are shown to illustrate the difference in signal, the random forest classifier used in this study is an algorithm that can discriminate even when linear discrimination is not possible. Therefore, it should be noted that the first principal component shown in the example does not directly represent the vector that contributes the most to the classification in the manuscript. The most contributed features for the discrimination in the four molecules in this study is Ip, as shown in Figure 6 in the manuscript.

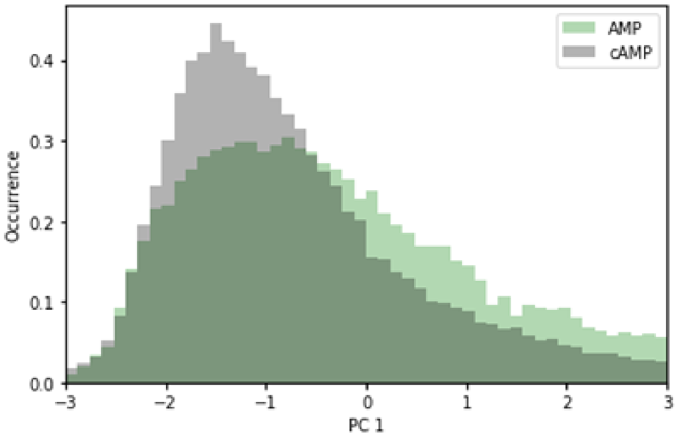

Figure S2. Distribution of the first component for AMP and cAMP. The first component is 0.29I_p + 0.05I_d + 0.09I_{ave} + 0.23S_1 + 0.30S_2 + 0.31S_3 + 0.31S_4 + 0.30S_5 + 0.30S_6 + 0.31S_7 + 0.30S_8 + 0.29S_9 + 0.33S_{10}.
